# The categorization of opaque pathologies outside of contrast media in hysterosalpingography which facilitate interpretation: A pictorial review

**DOI:** 10.18502/ijrm.v22i3.16163

**Published:** 2024-05-15

**Authors:** Fereshteh Hosseini, Fattaneh Pahlavan, Firoozeh Ahmadi

**Affiliations:** Department of Reproductive Imaging, Reproductive Biomedicine Research Center, Royan Institute for Reproductive Biomedicine, ACECR, Tehran, Iran.

**Keywords:** Hysterosalpingography, Opaque, Abnormalities, Uterine, Fallopian tubes.

## Abstract

Hysterosalpingography (HSG) is a practical and reliable imaging method to evaluate the cervical canal, uterine isthmus, uterine cavity, and fallopian tubes. Using HSG, opaque pathologies outside of contrast media can be detected as well as pathologies of uterus and fallopian tubes. We aim to present and categorize some uncommon and interesting abnormal findings that are located outside of the contrast areas in HSG. This is a pictorial review that depicts various types of HSG images that include opaque pathologies outside of the contrast areas. Images have been extracted from valuable archives collected over 50 yr by professor Shahrzad. A plain pelvic film contains soft tissues of the pelvis, bony structures, artifacts, or foreign bodies. Categorization might easily help the radiologist to interpret the HSG cliché. Opaque pathologies outside of contrast area in HSG can be categorized into 2 groups: “Pelvic Tissue Related” and “Foreign Bodies”. Pelvic tissue abnormalities might have a gynecologic or non-gynecologic source. Foreign bodies can be located in the pelvis or outside of the body. HSG is a reliable and inexpensive procedure. Familiarity with the pathologies of pelvic tissues and the accurate interpretation of HSG images are important.

## 1. Introduction

Several imaging methods are used to diagnose gynecological diseases and tumors. The first line would be an ultrasound examination with high sensitivity and specificity. In addition to ultrasound, magnetic resonance imaging has been used recently (1). It is a complementary imaging method, especially for the detection of cancers, pelvic endometriosis, pelvic floor disorders, indeterminate pelvic mass, and fibromas. However, it is an expensive method (2).

Laparoscopy and hysteroscopy are utilized for gynecologic assessment, infertility in particular. They are used since they have both treatment and diagnostic value; however, they are invasive (3). Hysterosalpingography (HSG) is a roentgenological visualization of the inner surface of the cervical canal, uterine isthmus, uterine cavity, and fallopian tubes. It is an inexpensive and non-invasive procedure (4). Not only is it a reliable method for the assessment of fallopian tube patency, but also it can detect intrauterine pathologies and some pelvic pathologies outside of contrast areas (5).

An in-depth investigation and interpretation of the opaque areas is therefore essential for obtaining reliable data. Pathologic findings should be followed up and ruled out using ultrasound examination and magnetic resonance imaging methods.

The pelvis consists of a complex compartment with multiple anatomic structures. Knowledge and attention to appearances of these structures and pathologic conditions are crucial to avoid confusion and preserve high diagnostic accuracy when interpreting pelvic findings. Radiologists should be conscious of possible nongynecologic findings in HSG (6). Plain pelvic radiographs contain the pelvic soft tissues, including the uterus, ovaries, muscles, and bladder, the gas patterns of the lower portion of the intestines, and bony structures such as pelvic bone, sacroiliac joints, the lower spine, and hips.

In a properly performed HSG, the endocervix, uterine cavity, and fallopian tubes can be well assessed. In addition, potential anomalies outside of contrast areas of the uterus, fallopian tubes, and other tissues may be seen and should be noticed. Soft tissue pathologies in HSG are visible if they calcify or form soft tissue masses (shadow of soft tissues) (7). HSG can also help evaluate the pathologies of bony structures (8). Puzzling features such as artifacts and foreign bodies in pelvic images might interfere with the interpretation.

This study aimed at presenting and evaluating rarely reported and abnormal opaque findings in HSG. The data were extracted from an archive collected over 50 yr. Images were obtained and sourced by Dr Shahrzad's imaging center and the collection were donated to the Department of Radiology, Royan Institute for Reproductive Biomedicine, Tehran, Iran.

## 2. Findings and categories

The opaque pathologies irrelevant to contrast material in HSG are categorized into 2 groups (Table I).

**Table 1:** Categorization of the opaque pathologies irrelevant to contrast material in HSG


**Pelvic tissue related**		**Foreign bodies**	
**1- Gynecologic **	**2- Non-gynecologic**	**1- Intra-pelvic foreign bodies**	**2- External foreign bodies**
- Calcifications of gynecologic organs - Soft tissue masses - Odd findings	- Calcifications of non-gynecologic organs - Pelvic bone anomalies	- Platinum prosthesis - Clips - Dislocated intrauterine device - Drugs - Swallowed object - Remained surgical instruments	- Objects related to the patient's clothes (zippers, clips, and pins)
HSG: Hysterosalpingography			

### Pelvic tissue related 

Pelvic tissue-related anomalies are 1- gynecologic-related or 2- non-gynecologic in type.

#### Gynecologic related 

The uterine cavity and fallopian tubes are visualized as opaque portions in HSG, which normally do not involve other gynecologic organs unless they change through calcification and the growth of new masses, making a soft tissue shadow.

**A. Calcified fibromas**

**Figure 1:**
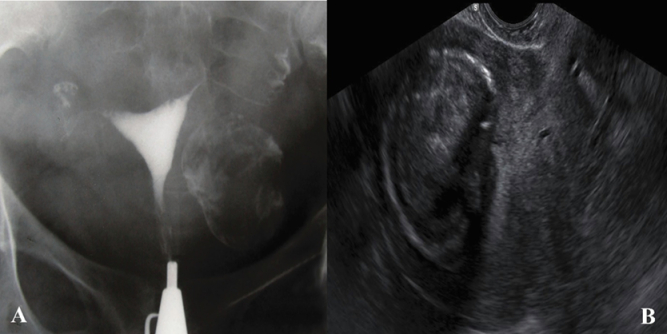
A) HSG of a 35-yr-old woman with primary infertility. The uterine cavity and fallopian tubes have a normal view. A calcified area is seen on the left side of the uterine cavity. B) The fibroma was approved by a 2-D ultrasound examination.

Uterine leiomyoma would not be detected on plain radiography unless the tumor has undergone calcified degeneration (9). This condition emerges as benign uterine masses with “mulberry” calcification patterns or a cyst wall appearing as rounded structures of varying sizes and locations (10) (Figure 1). Irregular coarse calcifications usually happen with subserosal leiomyomas, especially in tumors with pedicles (9).

**B. Calcified ovarian fibromas**

These calcified pelvic masses are rare tumors, but they may undergo dense calcifications. Ultrasound is required to rule out uterine fibroids and ovarian fibromas (11).

**C. Calcified ovarian tumor**

**Figure 2:**
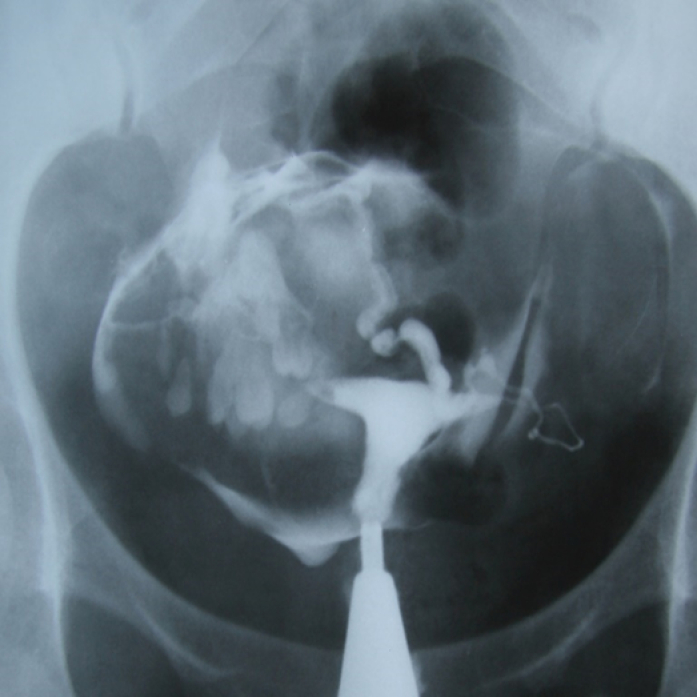
HSG of a 30-yr-old woman with secondary infertility, the uterine cavity is normal. A mild hydrosalpinx is evident at the end of the left tube. The right tube is displaced due to a soft tissue mass. Multiple dense areas with teeth structure appearance are seen inside the mass that represents a large dermoid cyst.

Ovarian calcification may have no specific cause or be associated with benign or malignant conditions such as endometriosis, serous neoplasms, mucinous lesions, teratoma, etc. One of the most common causes of ovarian calcification is dermoid cysts. Dermoid cysts emerge as high-density structures and the most prevalent ovarian tumors originate from primitive germ cell lines and contain hair, teeth, or nerves (Figure 2).

**D. Calcified fallopian tubes**

**Figure 3:**
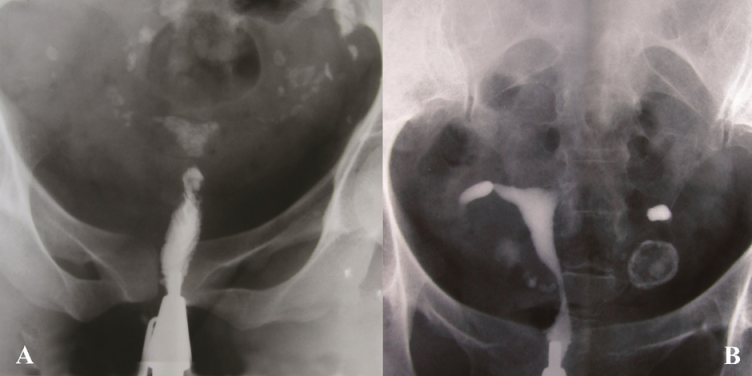
A) After injecting contrast materials, only the cervix was opaque in HSG. The uterus and fallopian tubes were calcified due to advanced TB infection. Calcified lymph nodes are also evident. Genital TB was approved by supplementary investigations. B) Normal uterine cavity. Obstruction in the middle portion of the isthmic segment of both fallopian tubes is seen. No peritoneal spillage was detected. The round calcified area on the left side of the pelvis denotes a calcified ovary caused by genital tuberculosis.

Secondary to tuberculosis: genital tuberculous can cause the calcification of the ovaries and fallopian tubes. The obstruction of the fallopian tubes might occur secondary to this pathology that is mostly bilateral (12) (Figure 3). Tubal calcification can take the form of linear streaks that lie in the course of the fallopian tube or may be seen as faint or dense tiny nodules. Tubal calcifications are usually small and may be straight, bent, or curved in shape.

**E. Soft tissue masses**

**Figure 4:**
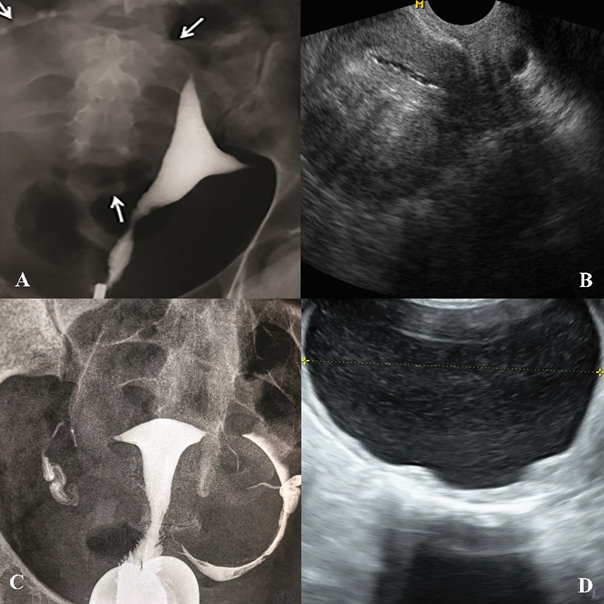
A) HSG of a 27-yr-old patient with primary infertility. A large soft tissue mass causing the elongation of the right wall and dislocation of the uterus to the left. B) An ultrasound image of patient “A”, which confirms the presence of a large fibroid. C) Dislocation of the left fallopian tube due to a soft tissue mass in HSG of a 37-yr-old patient. D) Ultrasound image of patient “C”, that shows an ovarian endometrioma cyst.

Frequently, a large soft-tissue mass is detectable in the hysterosalpingogram of the pelvis even if the mass is not calcified. The most common benign tumor of the genital tract which can be seen on a plain pelvic radiograph if it is large, is subserosal leiomyoma. It can be detected due to the distortion, dislocation, or deformation of the uterus and fallopian tubes (Figure 4).

**Figure 5:**
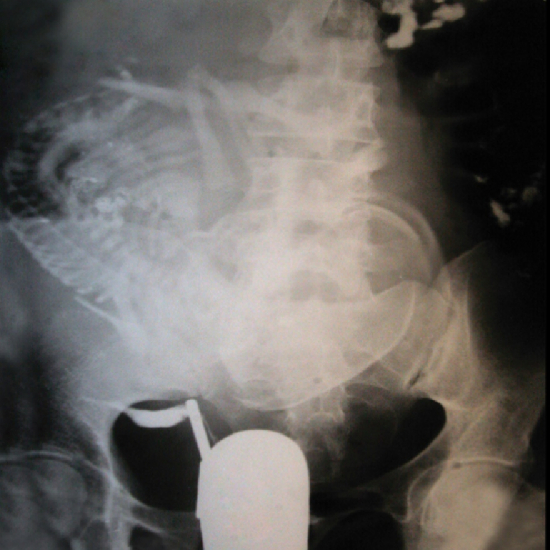
HSG of a patient referred with possibility of abdominal mass. (Taken 50 yr ago). An image of a fetus with overlapping cranial sutures is seen on the right portion of the abdomen and pelvis. The uterine cavity is visible on the right side of the pelvis. This finding suggests pregnancy on the left uterine cavity of the uterus didelphys.

In addition, a few odd and interesting findings might be seen in HSG. Figure 5 shows an astonishing finding of Professor Shahrzad's old collection. This image was taken 50 yr ago (before the development of ultrasound imaging) to assess abdominal and pelvic mass. The patient was referred to the hospital with a pelvic mass and an HSG had been done.

#### Non-gynecologic related

Particular attention to other pelvic structures is essential to determine the accurate diagnosis. Nongynecologic radiopaque changes in the pelvis can be related to the bowel and urinary tracts or localized to the peritoneal cavity or extraperitoneal pelvic structures (6). These conditions are not common but essential to recognize and distinguish from common gynecologic diseases.

**A. Calcifications of non-gynecologic organs**

**Figure 6:**
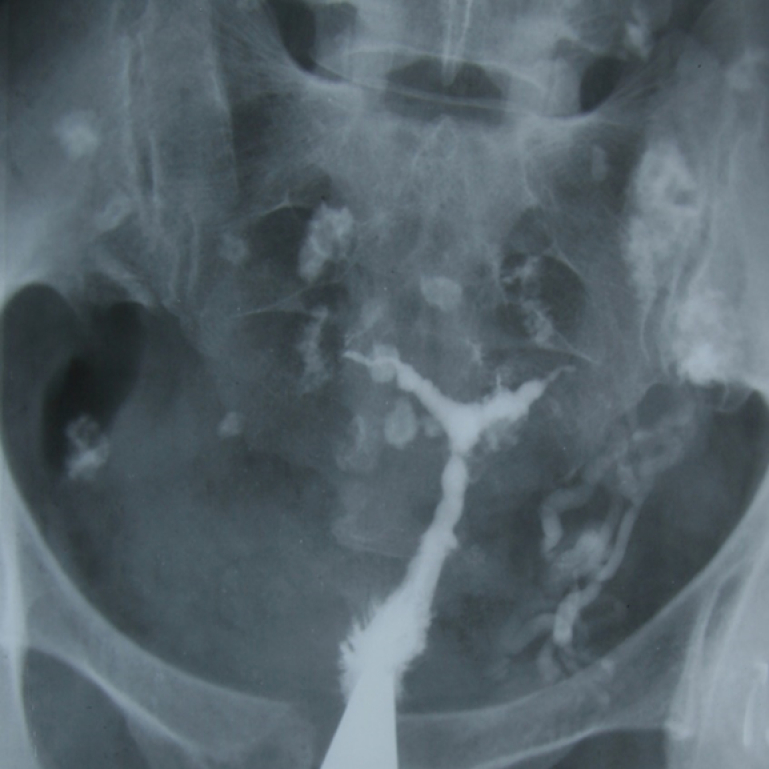
A 40-yr-old patient with secondary infertility. Uterine cavity with irregular borders and trifoliate appearance due to adhesions caused by genital tuberculosis. The calcified lymph nodes secondary to TB and intravasation are observed.

Calcified non-gynecologic organs and tissues might emerge as dense areas in HSG. The intestine, urinary system, lymphatic nodes, and vessels can develop calcified areas (13). Calcified mesenteric lymph nodes commonly appear as smooth, oval, and outlined structures in HSG because of infections, including tuberculosis (14) (Figure 6).

**Figure 7:**
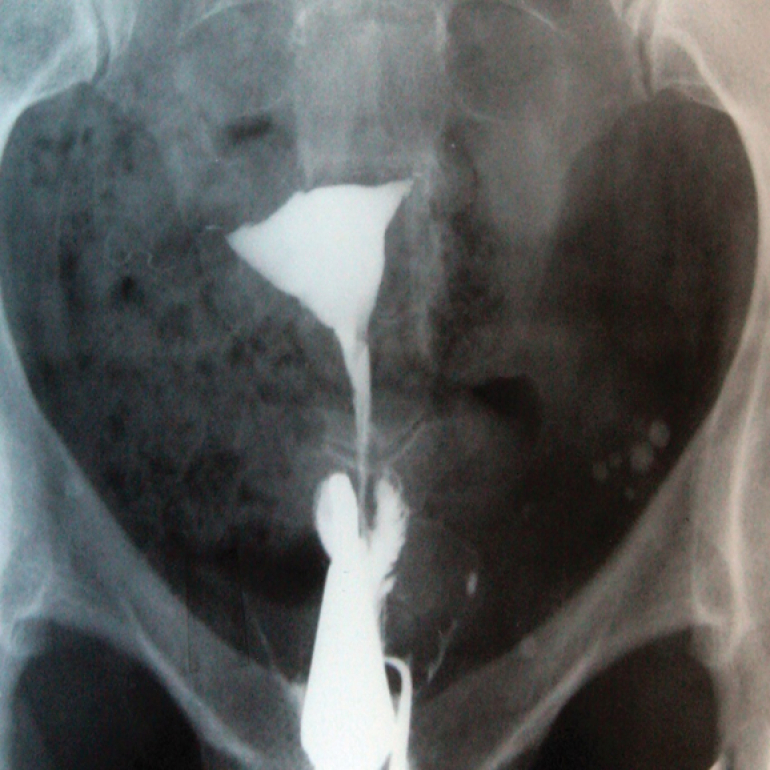
Normal uterine cavity. The concentration of tiny phleboliths on the left side of the pelvis and outside of the uterine cavity. Phleboliths are frequently seen in pelvic x-ray and have no clinical value.

Vascular calcification affects both arteries and veins. Pelvic phleboliths refer to the calcification of veins, emerging as vein stones, develop small, smooth, round, and calcified masses and mainly affect the lower half of the pelvic inlet (7) (Figure 7). As an incidental finding, this condition is of no pathological significance.

**Figure 8:**
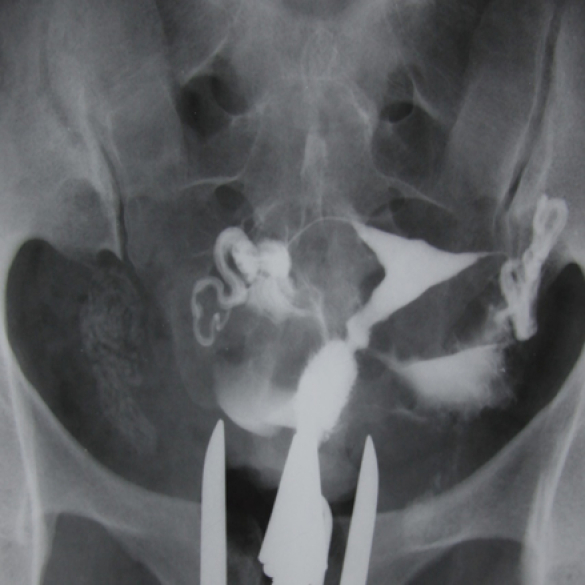
A shapeless calcified mass is seen on the right side of the pelvis in HSG. A calcified intestinal parasite was suspected based on the mass features and the patient's history. The patient was referred to an infectious disease specialist. After the medical laboratory test including a stool exam, the presence of the parasite was confirmed, and the patient was treated.

The intestine and its content make some distinct features such as calcified parasites (Figure 8).

**B. Pelvic bone anomalies**

**Figure 9:**
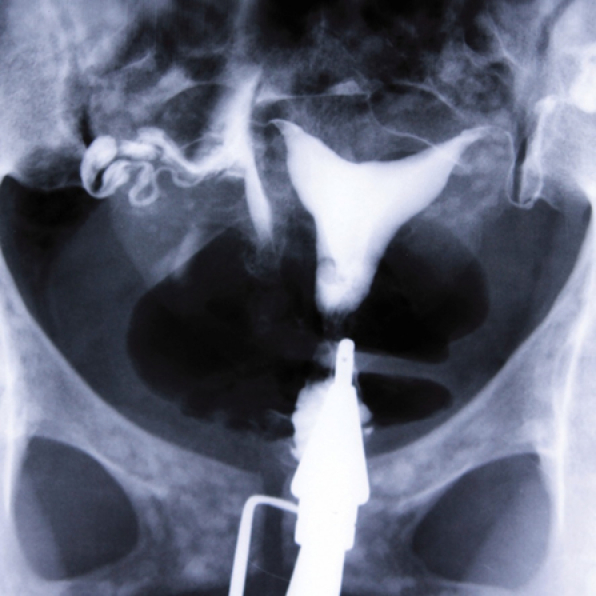
HSG of a 38-yr-old woman with secondary infertility. An arcuate uterus and normal fallopian tubes in HSG. Osteopoikilosis was diagnosed based on the small area of sclerotic bony lesions in the pelvic bones.

Bone lesions include damaged areas of bone due to infections, fractures, cysts, and tumors. Most bone lesions are benign. These lesions and diseases of bone tissue can be reflected in HSG. Therefore, assessment of bone tissue is necessary when an HSG cliché is reported (Figure 9).

### Foreign bodies

Retained radiopaque foreign bodies appearing in or out of the body in HSG are categorized as intra-pelvic foreign bodies or external bodies. Depending on the anatomical location, type of material, and size of the object, they may affect the evaluation of the intrapelvic organs.

#### Intra-pelvic foreign bodies

**Figure 10:**
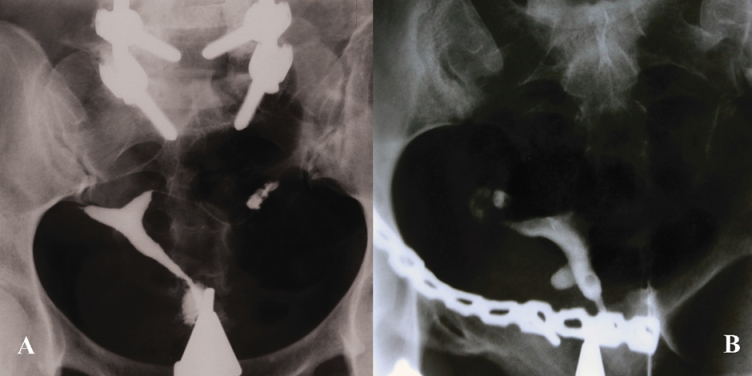
Platinum prosthesis. HSG of 2 different patients with primary infertility and history of surgery. A) A pin related to a previous orthopedic vertebral surgery. B) A chain associated with a pelvic surgery.

**Figure 11:**
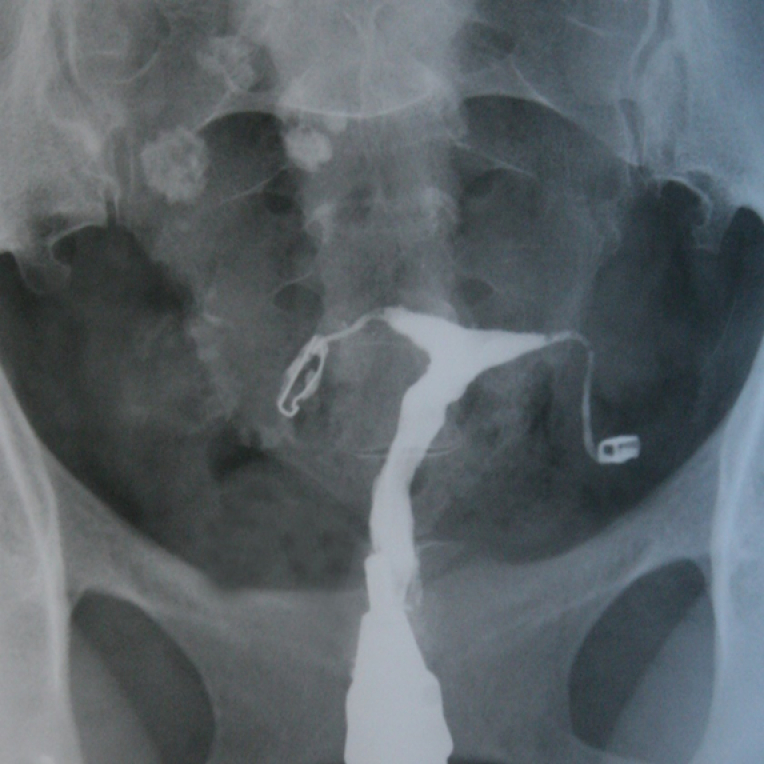
HSG of a 31-yr-old patient with primary infertility and a history of genital tuberculosis. 2 clips located in the middle of the fallopian tubes observed in HSG had been used to prevent the transmission of genitalia tuberculosis to the uterine cavity. Calcified lymph nodes secondary to TB are seen.

**Figure 12:**
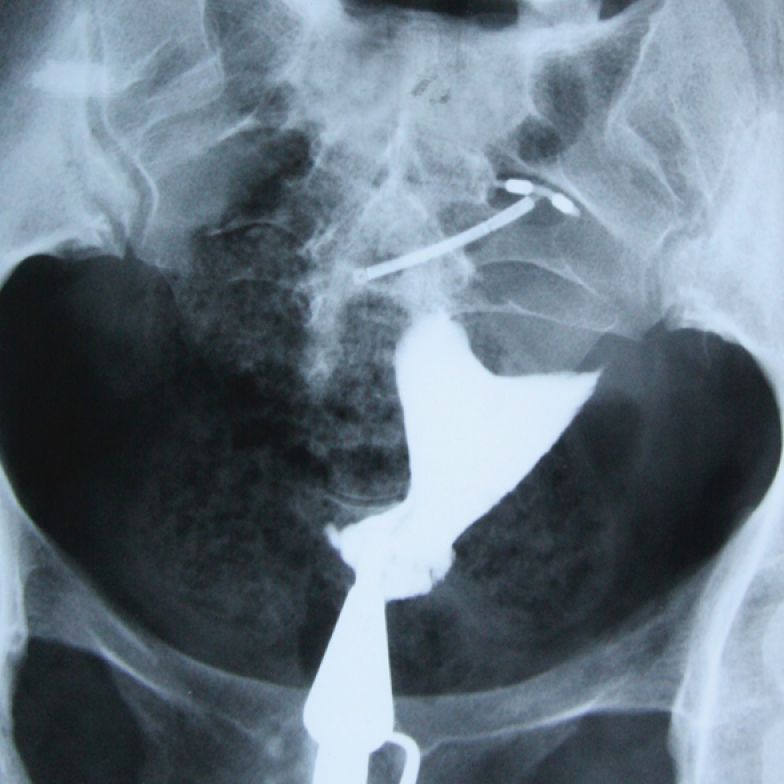
A dislocated IUD outside the uterine cavity in HSG. The patient has mentioned the history of putting in an IUD, but it was not detected during the examination by the gynecologist. After HSG, the patient underwent surgery, and the IUD was removed. (Patient thought that the IUD was already removed from her uterus).

**Figure 13:**
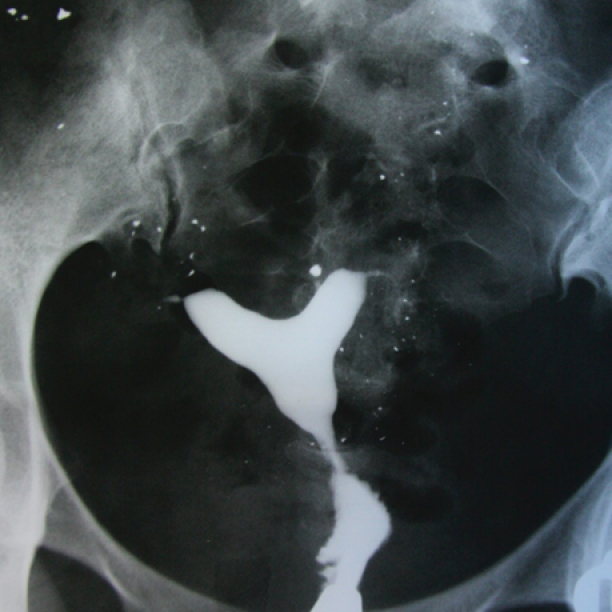
HSG of a 28-yr-old woman with primary infertility. A short fundal septum is evident. Bilateral tubal obstruction is seen. Medical powder (Bismuth) can be seen as scattered and fine granules or clumps through the bowel as observed in HSG. Tiny, dense, and diffuse light spots outside the uterus are caused by the opaque effect of Bismuth.

**Figure 14:**
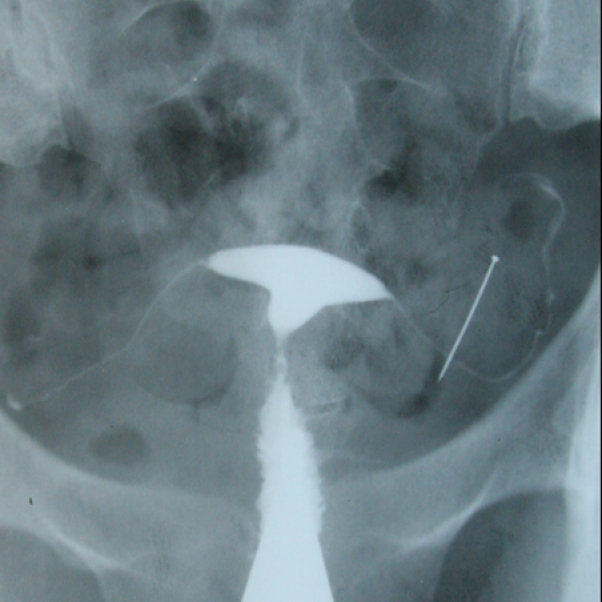
HSG of a patient with primary infertility. Cervix, uterine cavity, and fallopian tubes have normal view. Metal foreign body is seen on the left side of the uterine cavity in HSG. The patient had swallowed a needle. In complementary imaging assessment, no needle was detected after 3 days. It shows that the needle did not penetrate the soft tissue and has been excreted from the intestines.

**Figure 15:**
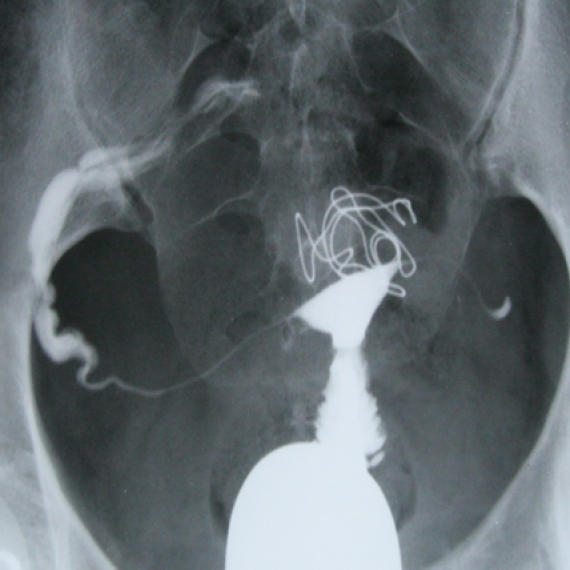
HSG of a patient with secondary infertility. Normal uterine cavity. The left fallopian tube failed to opacify but the right tube is filled and peritoneal spillage is observed. Retained surgical gas with radio-opaque markers above the uterine cavity is seen in HSG. The patient had pelvic surgery a few months ago; and referred for infertility work-up.

The radiopaque objects originating in the external environment may be introduced into the pelvic area through different ways. Intra-pelvic foreign bodies such as platinum prostheses, clips, dislocated IUD, drugs, needles, and retained surgical instruments or surgical packs can be medically located or inadvertently left in the pelvis by medical staff or be swallowed. The term “gossypiboma” or “textiloma" refers to a foreign object that is inadvertently left in body cavities after surgical operations. This condition is uncommon but not rare surgical error. Surgical instruments and devices are apparent in plain film because of the characteristic appearance of radiopaque tape or wires of laparotomy pads and surgical sponges, respectively. The retained surgical sponge may cause complications such as abdominal pains, nausea, vomiting, features of intestinal obstruction, or malabsorption syndrome (Figures 10–15).

#### External foreign bodies

**Figure 16:**
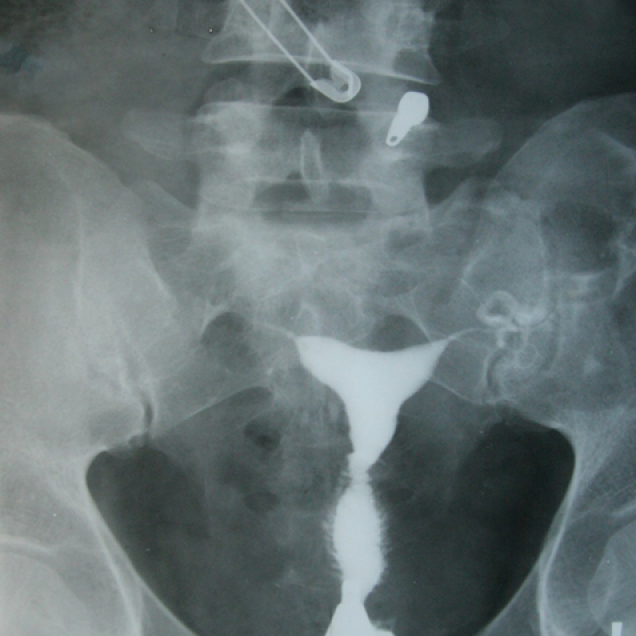
Normal uterine cavity and fallopian tubes. The safety pin and zipper attached to the patient's cloth are seen in the upper part of the HSG.

External bodies and objects related to patient clothes appearing as dense structures in HSG may interfere with the diagnosis of pelvic anomalies. Opaque objects in clothes or on the skin may appear to be within the pelvis (Figure 16). In case of ambiguity, patients should be covered with a sheet after taking off all their clothes.

## 3. Conclusion

Compared to other imaging and surgical procedures, HSG constitutes a reliable and cost-effective procedure for evaluating genital diseases. In addition to opaque areas showing the uterus and fallopian tubes patency, other soft tissues, pelvic bones, and their anomalies are visible in HSG.

Being familiar with the pathologies of pelvic tissues and accurately interpreting HSG images are therefore essential in identifying and treating gynecological diseases.

##  Data availability

Data supporting our findings can be sent by the co-author, upon request.

##  Author contributions

Firoozeh Ahmadi, Fattaneh Pahlavan, and Fereshteh Hosseini: participated in study design and data collection. Fattaneh Pahlavan and Fereshteh Hosseini wrote the manuscript. Firoozeh Ahmadi supervised the writing of manuscript. Fereshteh Hosseini collected and embedded images in the manuscript. All authors read and approved the final manuscript and take responsibility for the integrity of the data.

##  Conflict of Interest

The authors declare that there is no conflict of interest.
